# Regenerative Surgery of the Nonunion of Metacarpals and Phalanges: Amniotic Membrane and Bone Micro-Grafts as a Novel Treatment Approach

**DOI:** 10.3390/jcm14124024

**Published:** 2025-06-06

**Authors:** Francesco De Francesco, Andrea Marchesini, Michele Riccio

**Affiliations:** Department of Reconstructive Surgery and Hand Surgery, Azienda Ospedaliera Universitaria Delle Marche, 60126 Ancona, Italy; andrea.marchesini@ospedaliriuniti.marche.it (A.M.); michele.riccio@ospedaliriuniti.marche.it (M.R.)

**Keywords:** Rigenera^®^ technology, regenerative surgery, bone micro-grafts, amniotic membrane, nonunion, metacarpals, phalanges

## Abstract

**Background/Objectives**: Atrophic nonunion presents a significant challenge in hand surgery, often resulting in chronic pain and functional disability. Traditional surgical treatments such as bone grafting and internal fixation may be insufficient. This study evaluates the feasibility, safety, and preliminary effectiveness of a regenerative-first surgical protocol that combines autologous bone micro-grafts with a fresh human amniotic membrane to create a biologically active regenerative chamber. **Methods**: A total of 8 patients (6 males, 2 females; age range: 22–56 years) with an atrophic nonunion of metacarpals and phalanges were treated using a regenerative-first surgical approach. Autologous bone was harvested from the iliac crest and mechanically disaggregated via Rigenera^®^ technology to obtain micro-grafts enriched with osteoprogenitor cells and extracellular matrix fragments. These were applied to the bone defect and wrapped in a fresh amniotic membrane, creating a biologically active chamber. Fixation was achieved using low-profile plates or screws, and all patients underwent early protected mobilization. **Results**: Radiographic consolidation was achieved in all patients within 2 months postoperatively. Functional outcomes at final follow-up demonstrated excellent or good results in Total Active Motion (TAM), with grip and pinch strength within normative ranges and minimal residual pain. **Conclusions**: This preliminary series suggests that combining autologous bone micro-grafts with an amniotic membrane in a regenerative surgical protocol is a promising strategy for managing atrophic nonunion in the hand. The approach was associated with rapid consolidation and excellent functional recovery. Further research with larger, controlled cohorts is warranted to validate efficacy and define standardized indications and techniques.

## 1. Introduction

Atrophic nonunion of the metacarpals and phalanges is a challenging condition in hand surgery, characterized by an absence of biological healing activity and often resulting in persistent pain, limited hand function, and disability [1,2]. Conventional approaches such as autologous bone grafting and internal fixation are the mainstay of treatment [3,4,5], but in cases where the regenerative capacity of the host bone is severely impaired, outcomes may be suboptimal or even fail entirely [6,7]. Fractures of the metacarpals and phalanges are among the most common skeletal injuries, representing up to 40% of all hand fractures [8]. Although the majority heal uneventfully, nonunion remains a rare but clinically significant complication. The reported incidence of nonunion in these fractures ranges from 0.5% to 1.5% in modern series, with higher rates in cases involving segmental bone loss, open fractures, or infection [9]. Classic work by Jupiter and colleagues noted that nonunion affects both metacarpals and phalanges, with the phalangeal shaft showing an incidence of about 0.7% [10]. Current surgical strategies for managing these nonunions typically include autologous bone grafting, rigid internal fixation, or combinations thereof. However, such approaches may fail in biologically inactive or hypovascular environments. Delayed unions and nonunions often require revision surgeries that involve debridement, bone graft augmentation, and prolonged immobilization [11]. In some cases, soft tissue conditions and scar tissue further compromise outcomes, emphasizing the need for biologically supportive interventions that not only stabilize the fracture but also stimulate intrinsic bone healing.

Regenerative medicine has rapidly evolved as a cornerstone of translational biomedical research, particularly in the field of tissue repair and reconstruction. Rather than merely substituting damaged tissues, regenerative strategies aim to biologically stimulate endogenous repair mechanisms using stem cells, bioactive matrices, and growth factor delivery systems. In this context, De Francesco and colleagues have proposed a revision of the classic reconstructive ladder into what is now referred to as the “translational reconstructive scale”, where regenerative approaches such as cell-based therapies and bioengineered constructs are recognized as integral tools within the surgical decision-making process, especially in complex and biologically compromised cases [12]. This evolving framework allows clinicians to integrate biological treatments in a staged, patient-specific approach, moving beyond traditional mechanical reconstruction toward tissue bioactivation [13]. It has relevance in orthopedics and hand surgery, where the biological failure of bone healing—as seen in atrophic nonunion—requires interventions that not only restore structure but also reinitiate osteogenic activity within a favorable cellular and vascular microenvironment [14,15]. These strategies aim to mimic the physiological processes of bone repair—such as cellular recruitment, osteoinduction, and angiogenesis—thereby creating a microenvironment more conducive to effective and timely healing, particularly in cases where conventional treatments fail due to compromised biological activity [16,17].

Among these, autologous bone micro-grafts have garnered attention for their ability to deliver osteoprogenitor cells, growth factors, and extracellular matrix components directly to the site of nonunion. These micro-grafts are typically derived from autologous cancellous bone and processed intraoperatively using technologies like Rigenera^®^, which mechanically disaggregate the tissue while preserving the integrity of cellular and bioactive components essential for osteogenesis [18]. Studies in craniofacial and orthopedic models suggest that micro-grafts processed with Rigenera^®^ technology can enhance tissue regeneration and supporting bone healing processes [19,20,21,22,23]. Parallel to this, the application of amniotic membrane as a biological scaffold has demonstrated significant potential in orthopedics due to its anti-inflammatory, anti-fibrotic, and pro-angiogenic properties [24,25]. Derived from human placental tissue, the amniotic membrane acts as both a physical barrier and a bioactive matrix, creating a conducive environment for cell proliferation and vascularization while minimizing scar formation and immunologic response. Clinical applications have shown improved healing profiles and reduced complications when amniotic membranes are integrated into orthopedic and surgical wound treatments [26,27,28].

The convergence of these two biologically active strategies—bone micro-grafting and amniotic membrane scaffolding—offers a novel and synergistic approach to stimulate bone regeneration in refractory cases of atrophic nonunion. This study presents a regenerative surgical protocol based on the concept of a “biological regenerative chamber”, formed by autologous bone micro-grafts and an amniotic membrane, aimed at enhancing cellular recruitment, osteoinduction, and vascular integration. The primary objective is to evaluate the clinical feasibility, safety, and preliminary efficacy of this strategy in eight patients with long-standing, treatment-resistant nonunion of the metacarpals and phalanges, with the goal of accelerating graft integration and promoting faster functional recovery.

## 2. Materials and Methods

### 2.1. Study Design and Patient Selection

This study presents a retrospective observational series of 8 patients (6 males, 2 females; age range: 22–56 years) treated for atrophic nonunion of the metacarpals and phalanges, using a regenerative surgical protocol as a first-line reconstructive approach. Given the exploratory nature of this proof-of-concept study and the selected population of biologically compromised nonunion, no control group was included. The primary objective was to assess feasibility, safety, and preliminary effectiveness before planning larger controlled trials. In all cases, the decision to proceed with biological augmentation using autologous bone micro-grafts and amniotic membrane was made considering the poor biological conditions at the fracture site and the high risk of failure with standard reconstructive strategies, moreover, to fill the residual biological void following surgical debridement. Rather than attempting further traditional fixation or grafting procedures, a regenerative approach was prioritized to enhance osteogenic potential from the outset. All patients presented with persistent pain, functional limitation, and radiographic evidence of nonunion at least 4 months post-initial fracture treatment.

Inclusion criteria comprised radiologically confirmed atrophic nonunion, absence of active infection, and the presence of biological indicators of low healing capacity (e.g., minimal callus formation, osteolysis at the fracture site). Patients were selected based on clinical and radiological evaluation, with the intention to implement a regenerative-first strategy. The decision to adopt a regenerative-first approach was made in cases where conventional reconstructive strategies were deemed insufficient due to these biological limitations.

### 2.2. Surgical Technique

All procedures were performed under regional anesthesia and tourniquet control. Following standard antiseptic preparation, the nonunion site was surgically exposed. The fibrous and sclerotic tissue at the pseudarthrosis site was meticulously debrided until healthy, bleeding bone was encountered, in accordance with the biological principles of revitalization. To optimize the biological milieu, autologous cancellous bone was harvested intraoperatively from the iliac crest. The bone was mechanically disaggregated using the Rigenera^®^ system (HBW, Candiolo (TO), Italy), generating micro-grafts with a diameter of 80 μm. These micro-grafts are composed of clusters of viable osteoprogenitor cells, extracellular matrix fragments, and growth factors, and their size allows for diffusion-based metabolic support and rapid cellular proliferation at the recipient site. Given that the residual bone gap exceeded 1 cm, a corticospongious bone graft was employed to bridge the defect. The previously obtained micro-grafts were inserted within the corticospongious graft, enriching it with viable cellular and molecular components to enhance biological activity and osteoinductive potential. A fresh human amniotic membrane, certified by the Italian National Transplant Center (National Institute of Health, Roma, Italy) and provided by the Treviso Tissue Bank Foundation of Veneto (Treviso Hospital, Treviso, Italy), was then shaped to size and wrapped around the entire construct—the corticospongious graft loaded with micro-grafts—creating a “biological regenerative chamber.” This chamber provides both mechanical containment and a biologically active scaffold enriched in EGF, FGF, TGF-β, and PDGF, thereby enhancing osteoinduction, modulating inflammation, and supporting neovascularization. The membrane was secured with resorbable sutures or fibrin glue, depending on local anatomical constraints. Stabilization of the site was achieved via open reduction and internal fixation (ORIF) using low-profile plates or minifragment screws, chosen based on fracture type and anatomical constraints. The surgical goal was to restore mechanical stability while maximizing the regenerative potential through this dual biological strategy, with the aim of accelerating graft healing and promoting faster functional recovery.

In cases of proximal phalangeal nonunion with large bone loss, additional surgical strategies were implemented to minimize postoperative stiffness and preserve tendon gliding. Particular care was taken to avoid extensive periosteal stripping and to preserve the integrity of the extensor apparatus. When plate fixation was required, low-profile plates were positioned dorsolaterally rather than directly dorsal, to reduce the risk of extensor tendon impingement and lateral band adhesions. Furthermore, in all cases involving the proximal phalanx, we performed an extensive tenolysis of the extensor mechanism and lateral bands during surgical exposure, to release adhesions and restore tendon excursion.

### 2.3. Postoperative Protocol

All patients received prophylactic antibiotics for 48 h postoperatively. Immobilization was maintained for 2 weeks using a thermoplastic splint, followed by a gradual rehabilitation program focusing on active and passive range of motion exercises.

In cases with significant bone defects, particularly proximal phalangeal nonunions with large gaps (e.g., P4, P5, and P7), additional measures were implemented to optimize functional recovery. Early mobilization was prioritized, with controlled active range of motion exercises initiated as early as the second postoperative week, under close supervision. Customized dynamic splints were employed to facilitate tendon gliding while protecting the osteosynthesis construct, thereby minimizing the risk of extensor apparatus adhesions. Specific attention was given to scar management, edema control, and soft tissue mobilization, to prevent tethering of the lateral bands. Patients followed a structured rehabilitation protocol, including bi-weekly occupational therapy sessions, until satisfactory range of motion was achieved. Radiographs were taken at 4, 8, and 12 weeks postoperatively to assess callus formation and bone consolidation.

### 2.4. Clinical Outcome Assessment

Primary outcomes included radiographic evidence of bone union and absence of complications such as infection or graft failure. Radiographic union was defined as the presence of continuous bone trabeculae bridging at least three of four cortices in standard anteroposterior and oblique views. In addition to imaging, clinical consolidation was also assessed by evaluating the absence of pain upon palpation (digitopressure) at the fracture site, which is a recognized clinical sign of bone healing.

Secondary outcomes assessed functional recovery using range of motion (ROM), grip strength, and pain reduction measured by the Visual Analog Scale (VAS). DASH (Disabilities of the Arm, Shoulder, and Hand) scores were collected preoperatively and at final follow-up.

### 2.5. Radiographic Outcome Assessment

Radiographic evaluation of bone healing was performed at 1 and 2 months postoperatively using standardized anteroposterior and oblique views of the affected segment. Bone consolidation was defined as the radiographic presence of continuous bone trabeculae bridging at least three of four cortices in standard anteroposterior and oblique views, in line with established criteria in the fracture healing literature. Images were acquired using the same radiological equipment and stored in JPEG format via the hospital’s PACS system at 300 dpi. Each image was imported into Adobe Photoshop CC 2024 (version 25.12, Adobe Systems Inc., San Jose, CA, USA) for digital analysis. Grayscale analysis was performed on original images exported without applying post-processing filters, contrast, or brightness adjustments. All tonal values were extracted in their native form to preserve the original radiodensity information. Additionally, the use of the IRBH—calculated as a ratio between the defect area and adjacent healthy cortical bone within the same image—allowed internal normalization, minimizing variability due to radiographic acquisition settings. A Region of Interest (ROI) was manually defined over the bone defect and surrounding healthy cortical area using the magnetic selector tool. Histogram analysis was then performed to measure the grayscale tonal value (intensity in pixels) within the ROI, which reflects local bone mineral density. Both the Defect Tonal Value (absolute grayscale value within the defect area) and the Tonal Value of healthy cortical bone were recorded. To standardize measurements and account for technical variability (e.g., differences in radiographic exposure or acquisition parameters), we calculated the Index of Relative Bone Healing (IRBH), according to the formula originally described by De Francesco and colleagues in our previous work [29], to evaluate healing progression. The IRBH was selected as the primary quantitative parameter for assessing healing progression, as it provides a normalized measure of mineralization dynamics relative to healthy bone. Although raw Defect Tonal Values were collected, only IRBH values were used for statistical analysis to minimize confounding factors and improve inter-patient comparability. An increase in this index over time was interpreted as a surrogate marker of progressive mineralization and bone healing. All measurements were performed by two independent, blinded assessors, and any discrepancies were resolved by consensus. This semi-quantitative analysis was complemented by a qualitative assessment of bone continuity, callus formation, and bridging across cortices. Radiographic union was defined as the presence of continuous bone trabeculae bridging at least three of four cortices, in accordance with accepted definitions in the fracture healing literature.

### 2.6. Ethical Considerations

All patients provided written informed consent for treatment and inclusion in this study. The procedures were conducted in accordance with the ethical standards of the institutional and national research committees and with the 1964 Helsinki Declaration and its later amendments.

### 2.7. Statistical Analysis

Statistical analyses were performed using SPSS version 30.0. Continuous variables were described using means ± standard deviation and/or medians (with interquartile range) according to their distribution. The Wilcoxon signed-rank test for paired data was used to compare pre- and post-operative values (range of motion, grip strength, VAS, and DASH scores), with statistical significance set at *p* < 0.05. Correlations between variables were assessed using Spearman’s correlation coefficient. Statistical analyses were performed with a descriptive and exploratory aim, focusing on temporal trends of bone healing parameters within the cohort, without inferential claims to generalizability.

## 3. Results

All treated patients with the regenerative surgical protocol, combining autologous bone micro-grafts and amniotic membrane within a biological regenerative chamber, achieved complete radiographic bone consolidation within 1 to 2 months postoperatively. No intraoperative or postoperative complications, such as infection, graft failure, or immune reactions, were observed in any of the cases (Table 1).

**Figure 1:**
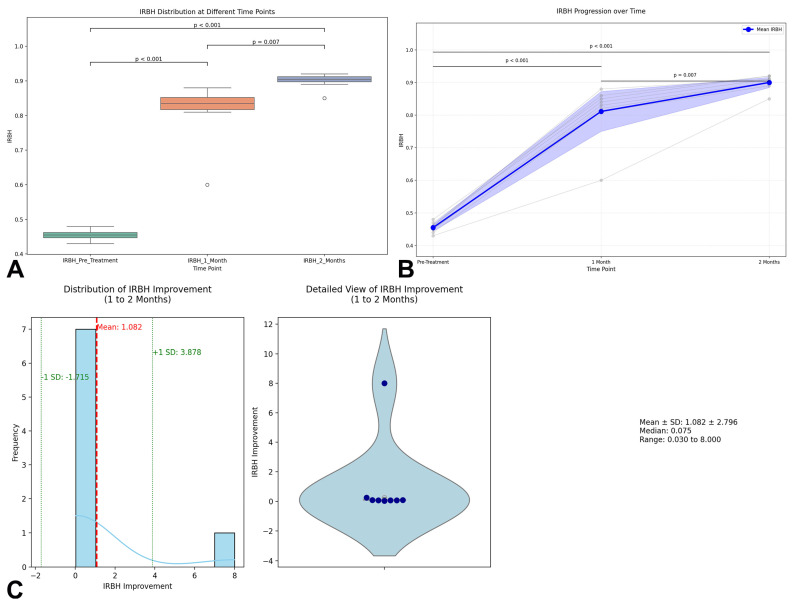
Quantitative evaluation of bone healing dynamics using the Index of Relative Bone Healing (IRBH): (**A**) Distribution of IRBH values at three timepoints. The figure displays the distribution of the Index of Relative Bone Healing (IRBH) at Pre-treatment, 1 Month, and 2 Months postoperatively, represented by boxplots showing median, quartiles, and outliers. The visual differences highlight the significant progressive increase in the IRBH over time, supporting the statistical tests performed. (**B**) Individual progression of the IRBH over time. The graph illustrates the evolution of the IRBH across the three evaluation points (Pre-Treatment, 1 Month, 2 Months), with individual patient trajectories represented by gray lines and the group mean with 95% confidence intervals depicted by a blue line. The *p*-values for pairwise comparisons (Pre-Treatment vs. 1 Month, 1 Month vs. 2 Months, and Pre-Treatment vs. 2 Months) are annotated within the figure through significance bars. (**C**) Pattern of IRBH improvement from 1 to 2 months. This composite figure presents two complementary visualizations: on the left, an enhanced histogram combined with a kernel density estimation (KDE) curve illustrates the distribution of IRBH improvement values, highlighting the mean and ±1 standard deviation limits; on the right, a combination of violin plot, boxplot, and swarm plot provides a detailed view of data distribution, including median, range, and dispersion. This integrated representation offers a comprehensive and immediate overview of the statistical variation in IRBH between the first and second postoperative months.

Radiographic evaluation of bone healing was quantitatively assessed through the Index of Relative Bone Healing (IRBH) at three timepoints: Pre-treatment, 1 Month, and 2 Months postoperatively. At baseline, the mean IRBH value was 0.455 ± 0.016 (range: 0.43–0.48), confirming poor mineralization and biological activity at the nonunion sites. One month after surgery, a significant increase was observed, with the mean IRBH rising to 0.811 ± 0.088 (range: 0.60–0.88), indicating an early progression of bone healing (*p* < 0.001). By the second month, the IRBH further improved to 0.900 ± 0.023 (range: 0.85–0.92), representing an additional significant enhancement compared to the first month (*p* = 0.007) (Figure 1A). The detailed IRBH values for each patient across the three timepoints are summarized in Table 2. The statistical analysis confirmed that the differences between all timepoints were highly significant: Pre-treatment vs. 1 Month: *p* < 0.001; 1 Month vs. 2 Months: *p* = 0.007; and Pre-treatment vs. 2 Months: *p* < 0.001. The individual progression trajectories of each patient are shown in Figure 1B, demonstrating a consistent and homogeneous trend towards healing across the cohort.

Notably, seven out of eight patients achieved radiographic consolidation within 2 months, characterized by cortical bridging across at least three cortices. Only one patient (P4), with a complex phalangeal nonunion, achieved complete consolidation at 3 months.

To further characterize the improvement in IRBH from 1 to 2 months, a detailed visualization was performed combining a histogram with kernel density estimation (KDE) and a violin plot integrating boxplot and swarm plot representations (Figure 1C). The distribution analysis showed a mean IRBH improvement of 0.089 ± 0.067 units during this interval, supporting the hypothesis of ongoing mineralization beyond the first month. The enhanced histogram highlights the mean improvement (red dashed line) and ±1 standard deviation bands (green dashed lines), providing additional clarity on data variability and distribution.

Overall, these results strongly support the regenerative approach’s efficacy in promoting bone healing, as demonstrated by the progressive and statistically significant increase in IRBH values across the postoperative timeline.

Functional outcomes measured using grip strength, pinch test (Jamar), and Total Active Motion (TAM) according to the Strickland classification, demonstrated significant improvement in all patients. At final follow-up, all subjects reported marked reduction in pain (VAS < 2) and the restoration of a nearly full range of motion. Clinical outcomes were rated as “excellent” and “good”, reflecting the rapid recovery and improved functionality attributed to the regenerative approach (Table 3).

**Figure 2:**
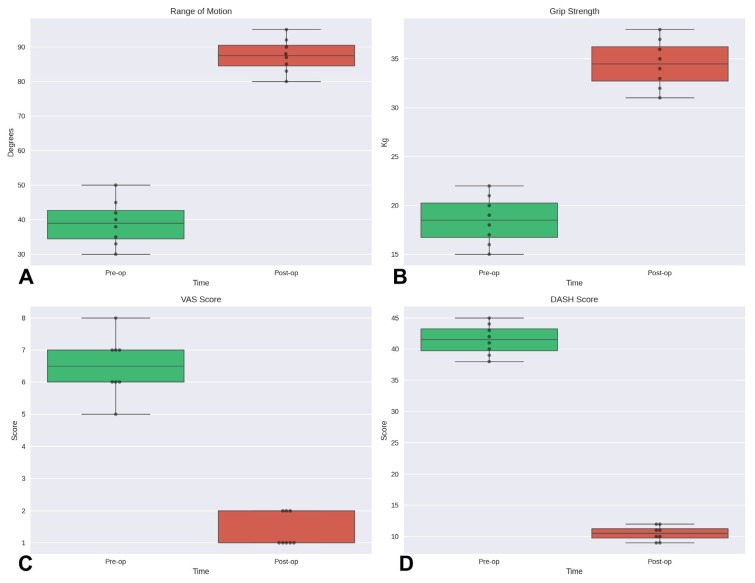
Boxplots with individual data points showing the distribution of pre- and post-operative outcomes: (**A**) Range of motion (degrees); (**B**) grip strength (kg); (**C**) Visual Analog Scale (VAS) for pain assessment; (**D**) Disabilities of the Arm, Shoulder, and Hand (DASH) score. Black dots represent individual patient data (*n* = 8). Boxes represent the interquartile range (25th to 75th percentiles), the horizontal line indicates the median, and whiskers extend to values within 1.5 times the interquartile range. Outliers beyond this range are shown as individual points.

In our analysis of data from the eight patients, statistically significant improvements were observed for all evaluated outcomes. Specifically, using the Wilcoxon signed-rank test for paired data, significant differences were demonstrated between pre- and post-operative values for range of motion, grip strength, VAS, and DASH scores (*p* < 0.05 for all comparisons) (Figure 2).

**Figure 3:**
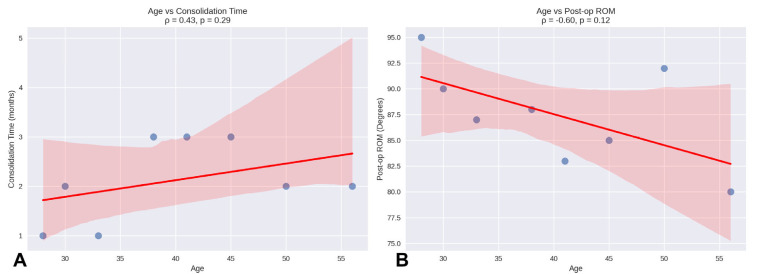
Correlation analysis between age and clinical outcomes: (**A**) Scatter plot showing the relationship between age and bone consolidation time (months) (ρ = 0.43, *p* = 0.29); (**B**) correlation between age and post-operative range of motion (degrees) (ρ = −0.60, *p* = 0.12). Red lines represent linear regression, and each point represents an individual patient (*n* = 8).

Regarding correlations, Spearman’s correlation analysis showed that the association between age and consolidation time (ρ = 0.43, *p* = 0.29) was not statistically significant. Similarly, the correlation between age and post-operative ROM was negative (ρ = –0.60, *p* = 0.12) and did not reach statistical significance (Figure 3).

### 3.1. Case 1

**Figure 4:**
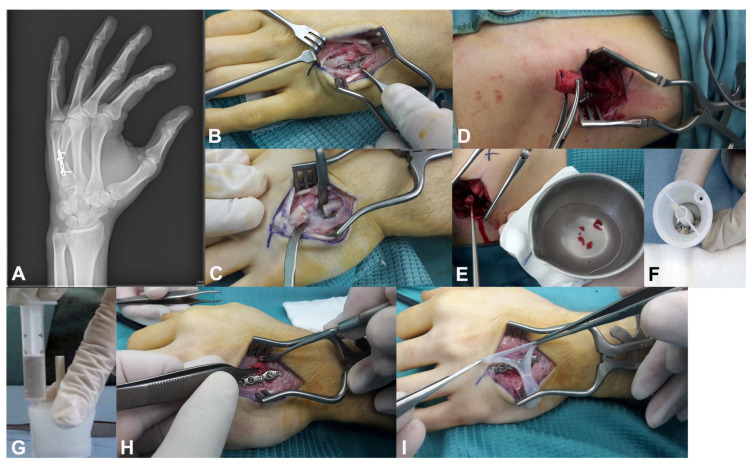
**Stepwise regenerative surgical treatment of an atrophic nonunion of the fourth metacarpal in a 22-year-old male**: (**A**) Preoperative radiograph showing an atrophic nonunion with sclerotic bone ends and absence of bridging callus. (**B**,**C**) Intraoperative debridement of the pseudarthrosis site exposing viable bleeding bone. (**D**–**G**) Autologous tricortical iliac crest bone graft harvested and mechanically disaggregated into micro-grafts using the Rigenera^®^ system; the graft bed is filled with the micro-graft suspension. (**G**–**I**) Application and microsurgical fixation of a fresh human amniotic membrane to create a sealed biological regenerative chamber over the graft and fixation site.

**Figure 5:**
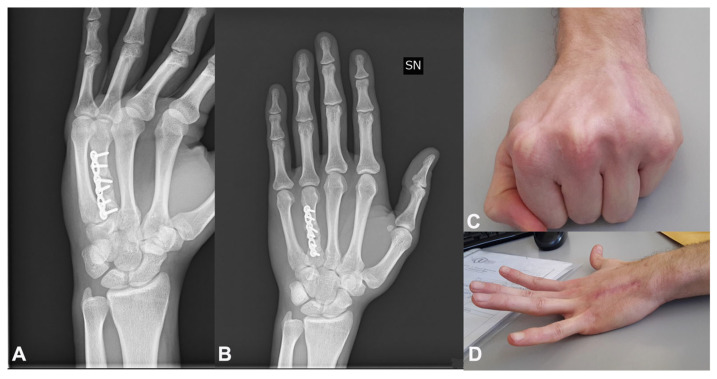
**Clinical and radiographic outcome at follow-up:** (**A**,**B**) Radiographs at 2 months postoperatively demonstrate complete bone consolidation of the fourth metacarpal with restoration of cortical continuity. (**C**,**D**) At 3 months, the patient exhibits full functional recovery with excellent TAM, grip strength, pinch strength, and minimal pain. No complications are observed.

A 22-year-old right-handed male manual worker presented with chronic pain and functional impairment of the dominant hand, seven months after sustaining a high-energy crush injury (P2). Initial management had consisted of the open reduction and internal fixation (ORIF) of a closed, comminuted fracture of the fourth metacarpal. However, despite surgery, the patient reported persistent pain and limited hand function, and radiographs confirmed an atrophic nonunion with sclerotic bone ends and the absence of bridging callus (Figure 4A). A regenerative surgical protocol was indicated. Surgical access was obtained through the previous dorsal incision. The nonunion site was thoroughly debrided until healthy bleeding bone was exposed (Figure 4B,C). A tricortical autologous bone graft was harvested from the iliac crest and mechanically disaggregated using the Rigenera^®^ system to produce viable cancellous micro-grafts enriched with osteoprogenitor cells and matrix fragments (Figure 4D–G). These micro-grafts were packed into the defect site and layered over the structural bone graft. A fresh human amniotic membrane was then trimmed to size and sutured using microsurgical stitches around the bone graft and fixation plate, creating a sealed biological regenerative chamber (Figure 4G–I). The fracture was stabilized using a low-profile dorsal plate, and a protocol of early protected mobilization was initiated postoperatively. At 2-month follow-up, radiographic evaluation confirmed complete bone consolidation. By 3 months, the patient had returned to full hand use with excellent functional recovery (Figure 5A–D). The patient exhibited a Total Active Motion (TAM) of 245 degrees, corresponding to a 94.2% recovery, thus indicating an “excellent” outcome as per the American Society for Surgery of the Hand (ASSH) criteria. Moreover, grip strength was 50 kg, pinch strength was 6 kg, and pain was minimal (VAS 1). No complications were reported. The patient expressed high satisfaction, and the clinical team considered the biological approach highly effective in restoring both structure and function.

### 3.2. Case 2

**Figure 6:**
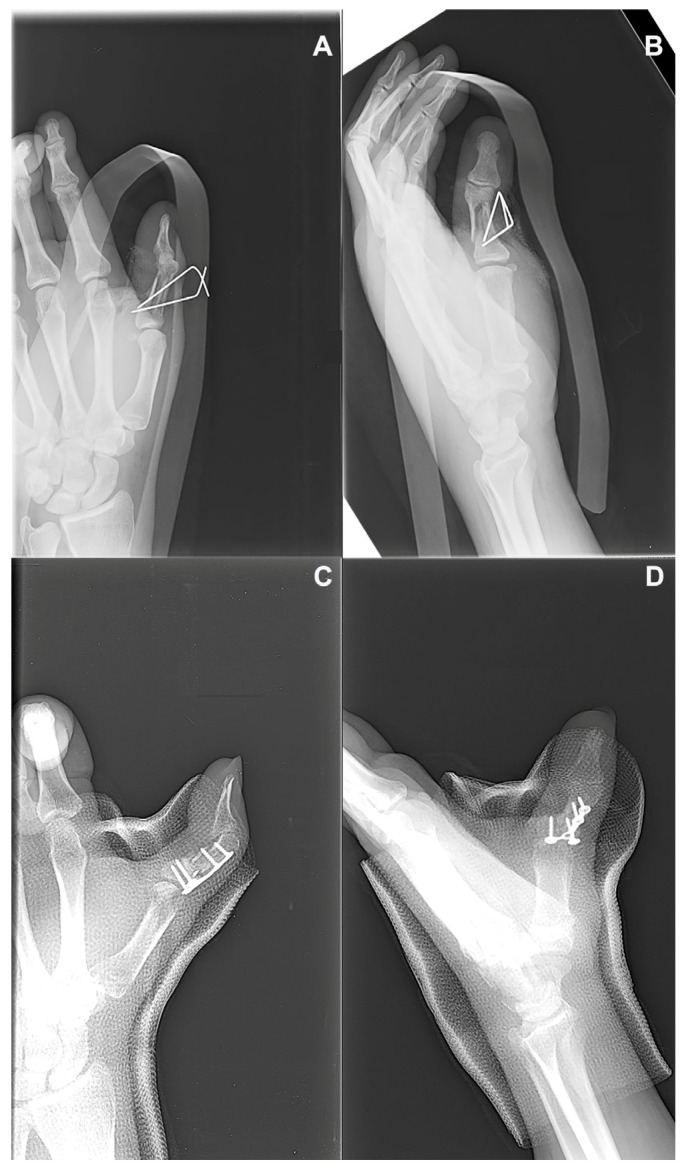
**Radiographic images of the third metacarpal fracture site:** (**A**,**B**) Preoperative anteroposterior and oblique views showing an atrophic nonunion characterized by the absence of bridging callus and sclerotic fracture margins, four months after initial fixation. (**C**,**D**) Postoperative radiographs obtained one month after the regenerative procedure, demonstrating progressive graft integration and early signs of fracture healing.

A 51-year-old right-handed male presented with persistent pain and limited functional capacity of the dominant hand, six months after a direct hand trauma that resulted in a diaphyseal fracture of the thumb phalanx (P8). The initial treatment had consisted of surgical fixation with Kirschner wires, but radiographs at follow-up showed an absence of bridging callus and sclerotic margins at the fracture site, consistent with an atrophic nonunion (Figure 6A,B). A regenerative strategy was undertaken as a first-line reconstructive approach, aimed at enhancing the biological environment. Surgical re-entry was performed through the original incision. The nonunion site was debrided meticulously to remove fibrous tissue and expose bleeding bone margins. A tricortical bone graft was harvested from the iliac crest and processed with the Rigenera^®^ system to obtain autologous cancellous micro-grafts. These micro-grafts, rich in viable osteoprogenitor cells and growth factors, were implanted into the nonunion site and integrated with the structural graft. Fixation was reinforced using a new low-profile plate to ensure stability during the osteointegration phase. A fresh amniotic membrane was rehydrated and wrapped around the grafted area, secured with microsurgical sutures to form a biological regenerative chamber. Postoperative management included early protected mobilization and edema control. After one postoperative month, radiographs confirmed complete consolidation (Figure 6C,D). At 3-month follow-up, the patient exhibited a Total Active Motion (TAM) of 110 degrees for the thumb, corresponding to an 84.6% recovery based on a theoretical maximum of 130°, thus indicating a “good” outcome according to the adapted American Society for Surgery of the Hand (ASSH) criteria for thumb function. A residual coronal plane deviation of 10 degrees and a fixed flexion contracture of 15 degrees at the proximal interphalangeal (PIP) joint were observed. Despite these findings, grip strength was 46 kg, pinch strength was 6 kg, and pain was nearly absent (VAS 1). No complications were recorded. Functional evaluation using the Buck-Gramcko method, adapted for the thumb, yielded an estimated score of 15/15, classifying the outcome as “excellent”. However, it is important to note that this score may underestimate certain functional limitations, such as minor joint deviation or specific activity-related deficits, which are not captured by this scoring system despite their clinical relevance. The outcome was rated as “good” by both patient and surgeon, with satisfactory functional recovery.

## 4. Discussion

Metacarpal and phalangeal fractures constitute approximately 10% of all skeletal injuries and remain a frequent reason for surgical intervention in hand trauma care [30]. Although most cases heal uneventfully, a small but significant number evolve into nonunion, particularly in the presence of open, comminuted fractures or inadequate biological response. The foundational philosophy proposed by Amadio and colleagues emphasizes that optimal outcomes in hand surgery derive from surgical precision, early functional rehabilitation, and strong patient compliance [31]. Within this model, stiffness is a concern, mobility an obsession, and early active mobilization a necessary tool—provided that mechanical stability is ensured.

Nonunion, as defined by several authors, is the symptomatic absence of radiographic healing beyond four months post-injury [2,9,10]. Its incidence ranges from 0.3% to 1% but may rise to 14% in high-risk groups, such as digital amputations, open fractures, or cases complicated by infection [32,33,34]. The conventional management of atrophic nonunion involves surgical debridement and corticospongious autograft stabilized with internal fixation (ORIF), particularly when the bone gap is under 2 cm [35]. While clinically accepted, this approach often entails protracted healing and modest functional recovery due to a lack of biological stimulation. Furthermore, the “graft critical point” posits that increasing graft volume may paradoxically hinder healing due to ischemia-induced necrosis and tissue fragmentation.

Mechanically disaggregated bone micro-grafts—such as those processed via Rigenera^®^ technology—offer an innovative solution to these challenges. Composed of 80 µm fragments containing viable osteoprogenitor cells and matrix elements, these micro-grafts allow nutrient diffusion and osteogenic activity without requiring immediate vascularization [19,20,21]. When combined with a structural autograft, they can enhance cellular signaling, osteoinduction, and bone remodeling. To complete the regenerative chamber, a human amniotic membrane is applied over the graft site. This membrane provides not only mechanical containment but also anti-inflammatory, angiogenic, and immunomodulatory effects, creating an ideal microenvironment for healing [36]. In the broader context of regenerative strategies for treating nonunion in hand orthopedics, several alternative methods have been explored. Bone marrow aspirate concentrate (BMAC) and platelet-rich plasma (PRP) are commonly used to enhance bone healing, but their efficacy is often limited by low progenitor cell yields and variability in clinical outcomes [37,38]. Mesenchymal stromal cells (MSCs) derived from adipose tissue or bone marrow offer more targeted regenerative potential, yet require complex processing, higher costs, and face significant regulatory hurdles [39]. Scaffold-based approaches, including demineralized bone matrix and synthetic bioceramics, provide osteoconductive support but lack the cellular and molecular components necessary for challenging biological deficits [40]. Compared to these methods, the Rigenera^®^ protocol allows for the intraoperative preparation of autologous micro-grafts enriched with viable osteoprogenitor cells and extracellular matrix fragments, with minimal manipulation and regulatory burden. This makes it a practical and biologically active solution for enhancing bone healing in clinical practice. Nevertheless, direct comparative studies are needed to evaluate the relative efficacy of these different regenerative approaches and define their specific indications. The idea of creating a biologically active chamber to promote bone regeneration has precedent in the “induced membrane technique” described by Masquelet. This two-stage approach involves the temporary placement of a PMMA spacer to stimulate the formation of a vascularized membrane, which subsequently hosts bone graft material [41,42,43]. While conceptually similar, our method differs in both timing and biology. The regenerative chamber is formed intraoperatively using native biological elements—bone micro-grafts and fresh amniotic membrane—without inducing a foreign–body reaction. This construct is biologically active from the outset, enabling single-stage reconstruction and early mobilization—advantages particularly relevant in hand surgery where functional recovery is time-sensitive.

Although the regenerative potential of mesenchymal stromal cells is widely recognized, their isolated application is insufficient for complete bone healing in complex scenarios. Regeneration requires the coordinated presence of mechanical stability, scaffolding structures, and bioactive factors. Our protocol integrates all these components—structural graft, micro-grafts, and a biologically active membrane—thereby addressing the multifactorial nature of bone regeneration. It is important to note that regenerative strategies do not eliminate the need for structural bone grafts in critical defects. Rather, they enhance graft integration and may reduce the required graft volume. Autologous corticospongious grafts remain essential as osteoconductive scaffolds, particularly in segmental defects where structural support is mandatory.

The clinical outcomes observed in our series are encouraging not only in terms of radiographic consolidation but also regarding the quality and timing of functional recovery. Bone healing was documented in all cases—significantly faster than the healing times typically reported in conventional nonunion treatment. The progression of IRBH values over time demonstrated a significant and consistent improvement, reflecting early biological activation followed by effective mineralization of the nonunion sites. Patients exhibited a homogeneous healing trend, with the majority achieving satisfactory cortical bridging within two months and only a minority requiring a slightly longer consolidation period. The distribution analysis of IRBH improvements between the first and second month confirmed ongoing biological activity beyond the initial healing phase, underlining the dynamic and progressive nature of bone regeneration under this protocol. These radiographic trends are in line with the observed clinical recovery and reinforce the notion that the combination of mechanical stability and biological stimulation provided by our regenerative approach can accelerate both structural and functional restoration.

Functionally, patients regained excellent or good hand performance: grip strength ranged from 25 to 50 kg, pinch strength from 3 to 8 kg, and Total Active Motion (TAM) was excellent in 75% of cases. Pain levels were minimal (VAS ≤ 2), and no complications, graft failures, or inflammatory responses were recorded, highlighting both efficacy and safety of the procedure.

These findings suggest that adopting a regenerative-first approach may be especially valuable in cases of biologically compromised nonunions. By enhancing the local osteogenic environment from the outset, this strategy could reduce the need for more invasive or staged reconstructive procedures and limit the duration of immobilization, which is often detrimental to hand function. The concept of the regenerative chamber introduced in this study embodies a synergistic combination of biological stimulation and mechanical stability. It provides a one-step alternative to the classical two-stage Masquelet technique, with the advantage of being immediately bioactive and more suitable for the smaller, functionally demanding anatomy of the hand. The integration of micro-grafts processed via Rigenera^®^ technology with a fresh amniotic membrane appears to create an optimal environment for bone regeneration. This synergy promotes angiogenesis, supports osteoinductive signaling, and facilitates matrix remodeling—critical processes for timely and effective bone healing. Importantly, in the context of hand surgery—where stiffness and functional decline are tightly correlated with delayed healing—protocols that accelerate bone consolidation while enabling early mobilization may represent a paradigm shift. They could significantly improve long-term outcomes by preserving joint function and allowing patients to resume daily activities more rapidly and safely. Nevertheless, it is important to emphasize that these findings represent preliminary evidence derived from a small case series. While the early consolidation outcomes are promising, definitive conclusions regarding efficacy and long-term benefits require validation through larger, controlled clinical trials.

These preliminary results suggest that integrating regenerative technologies into the treatment of biologically compromised nonunions could represent a paradigm shift in hand surgery. By accelerating bone healing and enabling earlier mobilization, this strategy may substantially enhance functional recovery, reduce the overall burden of care, and improve long-term patient outcomes.

This study has several limitations that should be acknowledged. First, the sample size was small (*n* = 8) and included heterogeneous fracture sites, limiting the generalizability of the results. Second, the retrospective, non-randomized design and the absence of a direct control group preclude definitive comparisons with standard treatments. The statistical analyses were limited to essential descriptive comparisons of bone healing dynamics over time, acknowledging the exploratory nature of this pilot study. Although the surgical procedures adhered to a common regenerative rationale, slight variations in graft volume, fixation technique, and rehabilitation protocol might have influenced individual outcomes. Moreover, the relatively short follow-up period was sufficient to assess bone consolidation but did not allow for the evaluation of long-term functional stability or graft durability. Additional barriers to the widespread adoption of regenerative protocols include elevated procedural costs, the limited availability of certified biomaterials, and regulatory restrictions surrounding cell-based therapies. Finally, larger, prospective, multicenter studies with anatomically homogeneous cohorts and extended follow-up are necessary to validate the reproducibility, long-term efficacy, and cost-effectiveness of this regenerative-first approach.

Future studies should focus on validating the regenerative chamber concept in larger, multicenter cohorts with anatomically homogeneous injuries. Comparative trials against standard techniques are needed to define the relative efficacy and cost-effectiveness of this approach. Furthermore, advancements in biomaterial engineering may further enhance the biological potential of next-generation regenerative protocols.

## 5. Conclusions

The results of this preliminary series suggest that the use of autologous bone micro-grafts in combination with a human amniotic membrane can provide a biologically active and mechanically stable solution for the treatment of atrophic nonunion in the hand. This regenerative approach appears to enhance bone healing and functional recovery while minimizing surgical morbidity and the risk of complications. However, the small sample size, the absence of a control group, and anatomical heterogeneity of the treated cases inherently limit the generalizability of these findings. Although the outcomes are promising, larger controlled studies are necessary to confirm long-term efficacy, define precise indications, and establish standardized surgical protocols. Overall, the regenerative surgery protocol presented here—based on a biological chamber formed by amniotic membrane, Rigenera^®^-processed micro-grafts, and autologous bone graft—appears capable of reducing consolidation time and significantly improving functional recovery in cases of atrophic nonunion. Based on these preliminary findings, a regenerative-first approach combining bone micro-grafts and amniotic membrane appears to be a promising strategy for accelerating bone healing and restoring hand function in challenging cases of atrophic nonunion.

## Figures and Tables

**Table 1:** Summary of treated patients.

Patient ID	Age	Fracture Site	Time from Trauma	Fixation Method	Bone Gap	Bone Consolidation	Complications
P1	45	5th Metacarpal	7 months	ORIF	1 cm	2 months	None
P2	22	4th Metacarpal	7 months	ORIF	1.2 cm	2 months	None
P3	40	3rd Metacarpal	4 months	ORIF	1.2 cm	2 months	None
P4	35	2nd Proximal Phalanx	6 months	ORIF	1.5 cm	3 months	None
P5	56	3rd Proximal Phalanx	4 months	ORIF	1 cm	1 month	None
P6	52	3rd Metacarpal	4 months	ORIF	1.5 cm	2 months	None
P7	48	4th Proximal Phalanx	5 months	ORIF	1 cm	2 months	None
P8	51	Thumb Phalanx	6 months	ORIF	1.2 cm	1 months	None

**Table 2:** Radiographic assessment of bone healing using the IRBH at different timepoints.

Patient ID	IRBH Pre-Treatment	IRBH 1 Month	IRBH 2 Months	Bone Consolidation
P1	0.45	0.82	0.9	2 months
P2	0.48	0.85	0.92	2 months
P3	0.47	0.84	0.91	2 months
P4	0.43	0.6	0.85	3 months
P5	0.46	0.88	0.91	1 month
P6	0.44	0.81	0.89	2 months
P7	0.45	0.83	0.9	2 months
P8	0.46	0.86	0.92	1 months

**Table 3:** Functional outcomes and clinical results at 3-month follow-up.

Patient ID	Grip Strength (Kg)	Pinch Strength (Kg)	TAM	VAS	Overall Outcome
P1	45	8	Good	1	Excellent
P2	50	6	Excellent	1	Excellent
P3	46	6	Excellent	1	Excellent
P4	48	7	Excellent	2	Excellent
P5	37	4	Excellent	1	Good
P6	46	7	Excellent	1	Excellent
P7	44	5	Good	1	Good
P8	25	3	Good	1	Good

## Data Availability

The authors confirm that the data supporting the findings of this study are available within this article.
